# Obituary

**Published:** 1854-01

**Authors:** 


					﻿THE AMERICAN SOCIETY OF DENTAL SURGEONS.
This time-honored association, at its last meeting, revised and amended its
constitution, and passed several important by-laws. A committee consisting of
Drs. J. D. White and . E. Townsend, were appointed to print the revised con-
stitution and by-laws. We have not yet met with this in print, but we under-
stand that the fee for admission is increased to §25, and we see the following
by-law reported : Resolved, “That any member of this Society, who shall extol
his own peculiar merits over a fellow practitioner in the public prints, or em-
ploy means of advertisement which may be regarded by this Society as lowering
its dignity or compromising its character, shall be impeached, suspended or ex-
pelled.” This is very similar in purport and phraseology to a resolution of the
Mississippi Association, adopted at its second annual meeting. We see, also
the following, reported as the XIII Article of the Constitution : “ Dental Ethics
—Any dentist, who shall procure a patent for a remedy or instrument of dental
surgery, or who deals in patent remedies or nostrums, shall be disqualified for
membership.” We see by an after decision of the Society, that its members
are allowed to use patented instruments. A distinction then exists between a
‘‘ .remedy or nostrum,” and an “ instrument.” It is, therefore, the’operation
and not the instrument, that is the remedy for the disease.
We cannot but express the regret that our venerated President, Dr. E. Parmly
should have felt constrained to decline the office any longer. We feel assured
that the Association partake in this regret. We hope he may still meet with
the Society, and that we shall have the pleasure to welcome him in the Queen
City at the next meeting. The Society, in its present presiding officer, Dr. E.
Townsend, will have a dignified, courteous and able President.
NEW YORK SOCIETY OF DENTAL SURGEONS.
This society held its last annual meeting on Tuesday, the 13th of Sept, last, in
New York. We see but a brief report of its proceedings in the September num-
ber of the Recorder. The following officers were elected lor the present year-
President, Martin K.Bridges; 1st Vice President, Benjamin Lord; 2d do. A
Johnson; Recording Secretary, Lemuel Covell; Corresponding Secretary, Chas.
C. Allen; Treasurer, Benj. J. Maguire; Librarian, E. F. Arnoux.
THE MISSISSIPPI VALLEY ASSOCIATION OF DENTAL BURGEONS.
It will be recollected by the members of this association, that the time for
holding its annual meeting has been changed to the third Wednesday of Febru-
ary. The society will meet at 10, A M., of Wednesday, 22d of February next,
at the Ohio College of Dental Surgery. We hope to see a full attendance.
OHIO DENTAL COLLEGE ASSOCIATION.
The annual meeting of this association takes place this year on Monday, 10
A. M., 20th of February next, at the Ohio College of Dental Surgery. The
commencement exercises of the College comes off on Wednesday evening. It
is hoped that the stockholders will generally attend, and we shall be happy to
see as many of the Profession present as can attend.
PAYMENTS FOR THE REGISTER.
In each number of the Register we receipt the amount paid in by each sub-
scriber during the quarter. By reference to this table, subscribers can see how
their account stands. If any mistake is made in this table we hope to be in-
formed of it, and it will be corrected. Remittances by mail will be at our risk.
Those who find their names not in this list during the year, will please recollect
that the printer must be paid.
OBITUARY.
We are pained to see announced the sudden and melancholy end of Dr. Josiah
Flagg, of Boston. The following notice we clip from one of our daily papers:
Boston, December 20.
Dr. Josiah Flagg,a well-known dentist of this city, jumped from a third story
window to the street, this morning, and was instantly killed. It is thought he
was insane, induced by a belief in the delusion called spiritualism.
RECEIPTS FOR THE REGISTER.
Dr, A. Berry, Covington, Ky.,Vol. 7, $2,00
Dr. E. L. Holmes, Lockport. Ji. Y.,
vol. 7........................... 2,00
Dr. M. Kelley, Bentonville, Ia.vol.7, 2,00
Dr. D. Watters, Center P. O., Mont-
gomery co., O., vols. 4, 5 and 6,. . 6,00
Dr. J. A. Kennicott,Chicago, Ill.,vol.
4, 5 and 6,....................—	. 6,00
Dr. Johnson, Indianopolis, la. vol. 7 2,00
Dr. L. Claggett, Georget’n, Ky. vol. 6, 2,00
Dr. M.L. Pierce, Madina P.O., Olean
co., N. Y-, vol. 7...............2,00
Dr. J. Taft, Xenia, O., vol. 6 and 7,... 4.00
Dr. G. Watt, Xenia, O., vol. 6 and 7,. 4,00
Dr. E. I. Poof, Fort Madison, Iowa,
vol. 7,......................... 2,00
Dr. B. A. Satterthwaite,Lima,O.,vol.72,U0
Dental Journals Received.
AMERICAN JOURNAL OF DENTAL SCIENCE, for October.
DENTAL NEWS LETTER
DENTAL RECORDER	“	“
DENTAL EXPOSITOR	“ May.
Medical Journals Received.
The SOUTHERN JOURNAL OF THE MEDICAL AND PHYSICAL SCIENCES.
The ST. LOUIS MEDICAL AND SURGICAL JOURNAL.
BOSTON MEDICAL AND SURGICAL JOURNAL.
CHARLESTON MEDICAL AND SURGICAL JOURNAL.
The MEDICAL CHRONICLE, or MONTREAL MONTHLY JOURNAL OF ME-
DICINE AND SURGERY.
OHIO MEDICAL JOURNAL.
WESTERN LANCET.
IOWA MEDICAL JOURNAL.
Baltimore College of Dental Surgery.
Fourteenth Session, lS5!8-554.
CHAPIN A. HARRIS, A. M., M. D„
Professor of Principles and Practice of Dental Surgery.
THOMAS E. BOND, Jr., A. M„ M. D.,
Professor of Special Pathology and Therapeutics.
WASHINGTON R. HANDY, M. D..
Professor of Anatomy and Physiology.
ALFRED A. BLANDY, M. D.,
Professor of Operative Dentistry.
PHILIP H. AUSTEN, A. M., M. D.,
Professor of Mechanical Dentistry.
REGINALD N. WRIGHT, A. M., M D.,
Lecturer on Chemistry and Metallurgy.
The regular course of Lectures will commence on the 1st of November and close the
1st of March. The Infirmary, Mechanical and dissecting rooms will be open on the 1st
Monday in October, during which month there Will be occasional lectures from each of
the Professors.
Tickets for the Course, $110. Dissecting Ticket, (optional), $10. Diploma Fee, $30.
Matriculation Fee, $5.
P. H. AUSTEN, Dean,
84 Sharp Street, Baltimore.
NINTH ANNUAL ANNOUNCEMENT
OF
THE MIO COLLEGE OF DENTIL SURGERF.
Session of 1853-54,
The regular course of lectures in this institution, commences the
first Monday of November, and closes on the third Wednesday of
February.
FACULTY.
JAMES TAYLOR, M. D., D. D. S.
Professor of the Principles and Practice of Dental Surgery.
THOMAS WOOD, M. D.,
Professor of Anatomy and Physiology.
J. B. SMITH, M. D.,
Professor of Pathology and Therapeutics.
JOHN ALLEN, D. D. S.,
Professor of Operative Dentistry.
H. R. SMITH, D. D. S.
Professor of Mechanical Dentistry.
G. WATT, M. D.,
Lecturer on Chemistry,
Tickets for the course, $100,00 ; Matriculating Ticket, 5,00 ;
Diploma Fee, $25,00. Good board can be had at from $2,50 to
$3,00 per week.	JAMES TAYLOR, Dean.
A Card to the Dental Profession.
7'lie undersigned would respectfully inform the members of the
Dental Profession, that they have perfected a plan of mounting
teeth on Atmospheric plates, which entirely overcomes all the diffi-
culties heretofore, to wit: the warping of plates and a variety of
other difficulties. For which they have obtained Letters Patent,
and will furnish office rights on such reasonable terms, that we
think no operator will mount a set of teeth without it, after knowing
its advantages over the old method.
Those residing in the following states and territories may be
supplied with office rights by J, K. Rickey, Keokuk, Iowa, to
wit.; New York. Pennsylvania, Indiana, Missouri, Illinois, Michi-
gan, Wisconsin, Louisiana, Texas, California, Maine, Vermont,
Rhode Island, South Carolina, Iowa, Florida, Minnesota, and Ore-
gan Territories. In the remaining Territories, by Creder & Wil-
liams of Lancaster. Ohio.
J. K. RICKEY, Keokuk, Iowa.
H. L. CRIDER & D. WILLIAMS, Lancaster, Ohio.
A Card to the Profession.
I have recently received letters, giving information that
there are those going about, claiming to be my agents for
vending my improved method of mounting teeth with con-
tinuous gums, who are unauthorized by me. I would therefore
inform the Profession, that my agents will be furnished with
the necessary documents from me to make their contracts
valid ;and agents should be required, in all cases where nego-
tiations are being made, to exhibit their credentials. I wish
to avoid litigation in all cases where it is possible, although it
has become necessary, in a few instances, to institute suits for
infringements. This has been rendered necessary with Dr.
Hunter, as well as a suit for libel.
Having found it necessary to take out letters patent to secure
the benefit of my improvement, I shall most assuredly defend
this right, that those who purchase may be protected in its use.
Jan. 1, 1851.	J. ALLEN.
Mentobal.
W. Z. REES Respectfully informs his friends and patrons, that
he has REMOVED his Manufactory to Sixth Street, between Wal-
nut and Vine, South side. A general assortment of DENTAL
and SURGICAL INSTRUMENTS always on hand and made to
order. Also a general assortment of Pearl Handles and Mir-
rors always on hand
				

